# Serum bilirubin is negatively associated with white blood cell count

**DOI:** 10.6061/clinics/2019/e775

**Published:** 2019-07-29

**Authors:** Li Zhang, Chunmei Zhang, Zhaowei Meng, Lu Gong, Chongjie Pang, Xiangxiang Liu, Qing Zhang, Qiyu Jia, Kun Song

**Affiliations:** I Department of Infectious Diseases, Tianjin Medical University General Hospital, Tianjin, P.R. China; II Department of Nuclear Medicine, Tianjin Medical University General Hospital, Tianjin, P.R. China; III Department of Health Management, Tianjin Medical University General Hospital, Tianjin, P.R. China

**Keywords:** Bilirubin, White Blood Cell (WBC), Sex

## Abstract

**OBJECTIVE:**

Bilirubin is considered an important antioxidant, anti-inflammatory factor and immunomodulator. The current investigation aimed to explore the association between bilirubin and white blood cell (WBC) count in a large Chinese cohort.

**METHODS:**

A total of 61091 participants (29259 males, 31832 females) were recruited from a Chinese tertiary hospital. Data were sorted by sex, and the association between bilirubin and WBC count was analyzed after dividing bilirubin levels into quartiles.

**RESULTS:**

Most parameters (including age, body mass index, systolic blood pressure, diastolic blood pressure, alanine aminotransferase, total bilirubin, blood urea nitrogen, creatinine, uric acid, triglycerides and WBC count) were significantly higher in men than in women. Bilirubin displayed significant negative relationships with most other measured variables. Linear logistic regression analysis further indicated their negative relationships. Females showed a significantly higher frequency of leucopenia than males. Significant associations of leucopenia with high bilirubin quartiles were shown in binary logistic regression models for both sexes, with a much closer association in men than in women. For instance, for men with bilirubin levels in quartile 4, the adjusted likelihood of leucopenia was 1.600-times higher than that of men with values in quartile 1. For women with bilirubin levels in quartile 4, the adjusted likelihood of leucopenia was 1.135-times higher than that of women with values in quartile 1.

**CONCLUSION:**

Bilirubin is negatively related to WBC count. Significant associations exist between leucopenia and high bilirubin quartiles, and these associations are more obvious in men than in women.

## INTRODUCTION

Bilirubin is generally considered to be an end-stage product of heme metabolism. However, it has now been suggested to possess a number of crucial properties for the human body. For instance, it is a potent antioxidant as well as an anti-inflammatory factor that is capable of scavenging various reactive oxygen species and free radicals 1,2, as well as counteracting oxidative stress 3,4. Many epidemiological studies have observed an inverse relationship between bilirubin and a number of pathological abnormalities, such as cardiovascular diseases 5, metabolic syndrome 6, dyslipidemia 7, and diabetes 8,9. However, we retrieved only two previous investigations studying the association between bilirubin and white blood cell (WBC) count, and there was some discordance 10,11. Tsai et al. 10 analyzed 2458 apparently healthy adults in Taiwan and found that a higher level of serum total bilirubin (TB) was associated with a lower WBC count, regardless of other classic cardiovascular risk factors. In the second paper, which was from Australia, Badrick et al. 11 analyzed two groups of individuals. After the removal of patients with an elevated WBC count and TB level, the community-living patients showed a negative correlation between the two, but the intensive care unit patients showed no significant relationship. In this study, we intended to systematically evaluate the relationship between TB level and WBC count in a large cohort of Chinese individuals, paying special attention to the sex differences in the relationship.

## METHODS

### Design and recruitment

A cross-sectional, community-based health check-up investigation was conducted in our hospital with the collaboration of a multidisciplinary team over a period of 10 years (recruitment was initiated in July 2007), the method of which was reported previously 12-25. Briefly, a questionnaire was sent to all the self-reported ostensibly healthy participants to complete. A blood sample was subsequently obtained for each person. To limit confounding factors, the following exclusion criteria were implemented: participants with histories of hematological, hepatic, renal, gastrointestinal, inflammatory, infectious, thyroidal, oncological or immunological diseases; subjects taking any medicine that might influence WBC count, TB, inflammation, infection or the immune system; WBC count not within the laboratory calibration reference range; a high level of TB (>40 μmol/L or 2.34 mg/dL); a high level of alanine aminotransferase (ALT) (>100 U/L); excessive alcohol consumption; and pregnancy. To fulfill the purpose of this particular investigation, we collected and compiled data during the period from September 2010 to September 2015. A total of 61091 eligible subjects (29259 males, 31832 females) with adequate data for analysis were included.

### Ethics

The ethical, methodological and protocol aspects of this study were approved by the institutional review board and ethics committee of Tianjin Medical University General Hospital. We confirmed that all methods in the current study were conducted in compliance with the relevant guidelines and regulations. Written consent was provided by all participants in this research.

### Measurements

Fasting blood tests and anthropometric measurements of all participants were conducted during their visits to our institution. Measurements of body height (BH) in centimeters, body weight (BW) in kilograms, and body mass index (BMI) by dividing BW (kilograms) by the square of BH (meters^2^) were performed. The determination of systolic blood pressure (SBP) and diastolic blood pressure (DBP) was performed by using a sphygmomanometer. Biochemical indicators included ALT, TB, blood urea nitrogen (BUN), creatinine (Cr), uric acid (UA), total cholesterol (TC), triglycerides (TGs), and fasting glucose (FG), which were determined by an autoanalyzer (Hitachi Corporation, Tokyo, Japan). WBC count was measured on a hemocytometer analyzer (Sysmex Corporation, Kobe, Japan).

The laboratory calibration references for the parameters were as follows: ALT 5-40 U/L, TB 3.4-20 μmol/L (0.20-1.17 mg/dL), BUN 1.7-8.3 mmol/L, Cr 44-115 μmol/L, TC 3.59-5.18 mmol/L, TGs 0.57-1.70 mmol/L, FG 3.6-5.8 mmol/L, and WBC count 4.0-9.5×10^9^/L.

### Statistics

Data from men and women were analyzed separately. First, an independent sample's t test was performed to measure differences in the indices. Pearson bivariate correlations were analyzed among TB and other parameters. Linear logistic regression analysis was performed to assess the independent relationship between WBC count and TB. Adjustments were performed for possible confounding factors, including age, BMI, SBP, DBP, TC, TGs and FG. Then, the TB concentration was divided into quartiles. The intergroup frequency differences in leucopenia were examined by the chi-square test. Crude and adjusted odds ratios (ORs) for leucopenia with 95% confidence intervals (CIs) were analyzed using binary logistic regression models. We conducted the statistical analyses with Statistical Package for Social Sciences software (SPSS version 17.0, Chicago, IL, USA). Significance was indicated by a *p*-value less than 0.05.

## RESULTS

### Characteristics of the participants according to sex

There were differences among the parameters with respect to sex (Table 1). Males were older than females. Most of the parameters, including age, BMI, SBP, DBP, ALT, TB, BUN, Cr, UA, TGs and WBCs, were significantly higher in males than in females. The TC concentration was significantly lower in males than in females.

### Correlations between TB and other key variables

TB demonstrated significant negative relationships with most of the other variables, including age, BMI, BUN, UA, TC, TGs, FG and WBCs in men, as well as age, BMI, SBP, DBP, BUN, TC, TGs, FG and WBCs in women (Table 2).

### Relationship between WBC count and TB determined by logistic regression analyses

Linear logistic regression analyses were conducted, and a negative relationship between WBC count and TB was determined by the following equations. For males, WBC count = 4.309 - 0.023×TB + 0.001×age + 0.042×BMI + 0.004×SBP - 0.002×DBP + 0.026×TC + 0.084×TGs + 0.003×FG. This equation indicated that each unit increase in TB would cause a 0.023 unit decrease in TB. For females, WBC count = 4.239 - 0.009×TB - 0.015×age + 0.052×BMI + 0.006×SBP - 0.003×DBP - 0.048×TC + 0.191×TGs + 0.042×FG. This equation indicated that each unit increase in TB would cause a 0.009 unit decrease in TB.

### Incidence of leucopenia according to TB quartile

TB quartiles were calculated, and the respective frequency of leucopenia (defined as WBC count <4.00×10^9^/L) was compared between sexes. Females showed a significantly higher overall frequency of leucopenia than males. Detailed incidences by TB quartiles also demonstrated the same pattern of differences between the sexes, and leucopenia frequency increased as the TB level increased (Table 3).

### Correlations of leucopenia with different TB quartiles according to sex

Binary logistic regression models were adopted to calculate the associations of leucopenia with different TB quartiles according to sex, using the lowest TB quartile as a reference (Table 4). Significant associations were demonstrated for leucopenia in the high TB quartiles for both sexes, with significantly closer associations in men than in women. The adjusted risks included age, BMI, SBP, DBP, TC, TGs and FG as covariates. Significant ORs were maintained for leucopenia in all high TB quartiles in males, while the associations with the third and fourth TB quartiles were maintained in females. For instance, for men with TB levels in quartile 4, the adjusted likelihood of leucopenia was 1.600-times higher than that of men with TB levels in quartile 1. For women with TB levels in quartile 4, the adjusted likelihood of leucopenia was 1.135-times higher than that of women with TB levels in quartile 1.

## DISCUSSION

In mammals, due to the activities of the heme oxygenase and biliverdin reductase enzymes, bilirubin is produced during the physiological breakdown of heme. Bilirubin is not water soluble and requires a series of metabolic reactions for its further excretion, beginning with its binding to albumin. For decades, bilirubin has been viewed as an excretory product and a potentially toxic metabolite of heme metabolism that does not exert physiological benefits in humans. In particular, hyperbilirubinemia is generally viewed as a negative phenomenon because infants with jaundice are associated with kernicterus, and adults with jaundice are the harbingers of hepatic failure. However, over the span of human evolution, the production pathway of bilirubin has been consistently conserved. The teleological supposition is that bilirubin plays unique roles with physiological importance 26. In fact, bilirubin has been discovered to possess beneficial effects for the human body at physiologic concentrations. For instance, bilirubin has demonstrated an astonishing potency to scavenge overproduced free radicals and can also exert anti-inflammatory functions and powerful immunosuppressive effects. In addition, it can produce direct effects upon cell signaling 1-4. For example, bilirubin has been proven to be more effective at protecting lipids from oxidation than other water-soluble antioxidants, such as glutathione 27. Serum bilirubin has also been demonstrated to be a major contributor to the total antioxidant capacity in blood plasma 28.

Systemic inflammation and oxidative stress are important mechanisms in the development of various metabolic abnormalities 4. The powerful antioxidant and anti-inflammatory capacities of bilirubin are reported to be the basis for protection against diseases such as cardiovascular diseases 5 and metabolic syndrome 6. In addition, the anti-inflammatory effect of bilirubin has been demonstrated in its protective role against rheumatoid arthritis 29 and colitis 28. Bilirubin has also been shown to be an immunomodulator, which makes bilirubin helpful in the treatment of diseases such as multiple sclerosis 30, lupus erythematosus 31, and autoimmune encephalomyelitis 32. Several molecular pathways have been identified to explain the above mechanisms, for example, the nuclear factor kappa B (NK-κB) pathway 33 and the extracellular signal-regulated kinase 1/2 (ERK1/2) pathway 34,35.

Only two previous investigations have studied the relationship between TB and WBC count 10,11; the results in the study by Tsai et al. 10 were generally in agreement with our research. The advantages of the current study included the large number of participants and the comprehensive and robust statistical analyses plus an emphasis on sex differences. In our opinion, the probable mechanism for the negative relationship between TB and WBC count could be protean and complicated 10. First, negative correlations between TB and inflammatory markers are reported to exist, which could regulate WBC production 36. Second, an elevated WBC count could reflect enhanced cellular oxidative stress, which could lead to the consumption and even depletion of natural antioxidants, thus leading to a decrease in TB concentration 10. Third, metabolic abnormalities, such as metabolic syndrome, may be an important underlying link in the association between TB and WBC count. It is reported that people with an increased level of WBCs will have an elevated risk of metabolic syndrome development, which is possibly due to chronic inflammation 19. However, epidemiologic surveys have reported that TB was negatively correlated with a number of abnormalities, such as cardiovascular diseases 5 and metabolic syndrome 6. The proposed reason for this phenomenon is a regulatory effect derived from insulin resistance, which is the core proposed mechanism in metabolic syndrome pathogenesis 37. Finally, inflammatory responses could be suppressed by bilirubin due to its preventive effects on the migration of leukocytes into target tissues, which may be mediated by a disruption in vascular cell adhesion molecule-1-dependent cell signaling. Therefore, for example, bilirubin can prevent dextran sodium sulfate-induced colitis by inhibiting leukocyte migration across the vascular endothelium and by suppressing inducible nitric oxide synthase expression 38.

There are several limitations to our study. First, this study was cross-sectional, which does not allow for conclusions regarding causal relationships. Prospective and interventional investigations should be planned in the future. Second, we did not measure markers of inflammation in our population because of the budget shortage. Third, we measured blood parameters in only a single blood sample, and we did not confirm the results due to the budget shortage, which may have resulted in less-precise results than those obtained from repeated measurements. Fourth, serum TB may be influenced by a number of hereditary factors 39 or dietary habits 40, which were not fully analyzed in the current study. Finally, a number of the participants with various undetected confounding factors might not be aware of their medical conditions, which could influence our results.

## CONCLUSIONS

In conclusion, TB is inversely related to WBC count. High TB quartiles are associated with significant risks for leucopenia, and this risk was more obvious in men than in women. It seems reasonable to suggest the assessment of WBC count when abnormal TB levels are found. The exact reason behind this association still requires further investigation.

## AUTHOR CONTRIBUTIONS

Meng Z, Gong L and Zhang Q designed the investigation. Zhang L, Zhang C, Meng Z, Pang C, Liu X, Jia Q and Song K conducted the investigation and collected the data. Zhang L, Zhang C and Meng Z performed the statistical analysis. Zhang L, Zhang C, Meng Z and Pang C wrote the main manuscript. All authors reviewed and edited the manuscript and approved the final version of the manuscript.

## Figures and Tables

**Table 1:** Participant characteristics.

Parameter	Males	Females	T value
Case number	29259	31832	
Age (years)	49.10±12.56	47.45±13.06	15.824**
BMI (kg/m^2^)	25.68±3.18	23.97±3.47	62.901**
SBP (mmHg)	125.57±16.81	121.13±18.24	31.271**
DBP (mmHg)	80.48±11.17	74.70±10.26	66.748**
ALT (U/L)	25.06±13.61	18.41±10.69	67.438**
TB (μmol/L)	13.37±5.32	11.14±4.52	55.943**
BUN (mmol/L)	5.09±1.27	4.41±1.22	67.262**
Cr (μmol/L)	78.73±11.64	59.79±9.58	220.320**
UA (μmol/L)	357.77±74.99	263.93±59.84	171.599**
TC (mmol/L)	5.10±0.94	5.22±1.03	-14.718**
TGs (mmol/L)	1.73±1.31	1.27±0.86	51.581**
FG (mmol/L)	5.35±1.22	5.06±0.93	33.806**
WBCs (×10^9^/L)	5.72±1.18	5.38±1.11	37.084**

BMI=body mass index, SBP=systolic blood pressure, DBP=diastolic blood pressure, ALT=alanine aminotransferase, TB=total bilirubin, BUN=blood urea nitrogen, Cr=creatinine, UA=uric acid, TC=total cholesterol, TGs=triglycerides, FG=fasting glucose, WBCs=white blood cells.

** *p*<0.01 (analyzed by the independent sample’s t test).

**Table 2:** Pearson bivariate correlations between TB and other variables according to sex.

Parameter	Correlation coefficients for males	Correlation coefficients for females
Age	-0.016^**^	-0.012*
BMI	-0.059^**^	-0.081^**^
SBP	-0.003	-0.018^**^
DBP	0.002	-0.013*
ALT	0.007	0.025^**^
BUN	-0.060^**^	-0.036^**^
Cr	0.044^**^	0.041^**^
UA	-0.016^**^	0.005
TC	-0.046^**^	-0.020^**^
TGs	-0.100^**^	-0.069^**^
FG	-0.058^**^	-0.061^**^
WBCs	-0.123^**^	-0.059^**^

TB=total bilirubin, BMI=body mass index, SBP=systolic blood pressure, DBP=diastolic blood pressure, ALT=alanine aminotransferase, BUN=blood urea nitrogen, Cr=creatinine, UA=uric acid, TC=total cholesterol, TGs=triglycerides, FG=fasting glucose, WBCs=white blood cells.

* *p*<0.05, **p<0.01.

**Table 3:** Incidence of leucopenia in different sex by TB quartiles.

	Incidence (number of patients) in different quartiles				
	Quartile 1	Quartile 2	Quartile 3	Quartile 4	Total
Male					
Mean±SD (×109/L)	5.94±1.22	5.74±1.17	5.65±1.17	5.54±1.12	5.72±1.18
Leucopenia #	2.971% (220)	3.945% (291)	4.550% (326)	5.223% (382)	4.166% (1219)
Normal WBC #	97.029% (7184)	96.055% (7085)	95.450% (6839)	94.777% (6932)	95.834% (28040)
Female					
Mean±SD (×109/L)	5.49±1.15	5.38±1.10	5.34±1.10	5.29±1.08	5.38±1.11
Leucopenia #	6.968% (565)	7.930% (626)	8.529% (682)	8.745% (685)	8.036% (2558)
Normal WBC #	93.032% (7544)	92.070% (7268)	91.471% (7314)	91.255% (7148)	91.964% (29274)
Chi-square value					
Leucopenia comparison #	128.650**	107.267**	96.413**	71.659**	393.593**

TB=total bilirubin, WBC=white blood cell, SD=standard deviation.

# Leucopenia was defined as a WBC count <4.00×10^9^/L.

ˆComparing the incidence of leucopenia between males and females by the chi-square method.

** *p*<0.01.

**Table 4:** The risks of leucopenia according to TB quartiles in different sex.

TB Quartile	Males			Females		
	TB values	Crude OR (CI)#	Adjusted OR (CI)ˆ	TB values	Crude OR (CI)#	Adjusted OR (CI)ˆ
TB Quartile 1	TB <9.60 (μmol/L, reference)			TB <8.00 (μmol/L, reference)		
TB Quartile 2	9.60 ≤ TB <12.50	1.341 (1.122-1.603)^**^	1.285 (1.074-1.537)^**^	8.00 ≤ TB <10.30	1.150 (1.022-1.294)*	1.088 (0.965-1.226)
TB Quartile 3	12.50 ≤ TB <16.10	1.557 (1.308-1.853)^**^	1.467 (1.231-1.748)^**^	10.30 ≤ TB <13.40	1.245 (1.109-1.398)^**^	1.137 (1.011-1.279)*
TB Quartile 4	TB ≥16.10	1.799 (1.519-2.131)^**^	1.600 (1.349-1.898)^**^	TB ≥13.40	1.280 (1.139-1.437)^**^	1.135 (1.009-1. 277)*

TB=total bilirubin, OR=odds ratio, CI=confidence interval.

# Logistic regression model using TB Quartile 1 as a reference, including no covariate.

ˆ Logistic regression model using TB Quartile 1 as a reference, including age, body mass index, blood pressure, total cholesterol, triglycerides and fasting glucose ascovariates.

* *p*<0.05, **p<0.01.
